# Adverse Events in Isotretinoin Therapy: A Single-Arm Meta-Analysis

**DOI:** 10.3390/ijerph19116463

**Published:** 2022-05-26

**Authors:** Jan Kapała, Julia Lewandowska, Waldemar Placek, Agnieszka Owczarczyk-Saczonek

**Affiliations:** 1 Medical Faculty, University of Warmia and Mazury in Olsztyn, 10-719 Olsztyn, Poland; julia.lewandowska.1@student.uwm.edu.pl; 2 Department of Dermatology Sexually Transmitted Diseases and Clinical Immunology, University of Warmia and Mazury in Olsztyn, 10-719 Olsztyn, Poland; w.placek@wp.pl (W.P.); agnieszka.owczarczyk@uwm.edu.pl (A.O.-S.)

**Keywords:** isotretinoin, acne vulgaris, adverse events, single-arm meta-analysis

## Abstract

Isotretinoin (ISO) is an oral prescription-only retinoid, well known for its acne-treating effect. However, it affects a substantial number of human cell types, causing a broad spectrum of adverse effects. The purpose of this study is to establish the isotretinoin therapy adverse events among human clinical trials and their prevalence. Two authors (J.K., J.L.) systematically performed the literature review and assessment from December 2021–February 2022. Three databases (PubMed, ClinicalTrials, and Cochrane Library) were searched using the following terms: “isotretinoin acne vulgaris” for published studies in English from 1980–2021. Finally, 25 randomized controlled clinical trials (RCTs) and five open-label clinical trials provided 3274 acne vulgaris suffering patients. Isotretinoin therapy affects almost all of the systems in the human body, causing numerous adverse events. However, they mainly concern mild mucocutaneous conditions (severe cases are rare) and represent individual responses to a drug. In addition, all adverse events are reversible and can be avoided by specific preparations.

## 1. Introduction

Isotretinoin (ISO, (2Z,4E,6E,8E)-3,7-dimethyl-9-(2,6,6-trimethyl cyclohexen-1-yl)nona-2,4,6,8-tetraenoic acid), also known as 13-cis retinoic acid, is an oral prescription retinoid and vitamin A derivative in the form of a capsule that is the only approved medication to treat severe, resistant, nodular, and unresponsive to conventional therapy acne (Table 1). Other off-label indications not approved by the United States Food and Drug Administration (FDA) for use include moderate acne, cutaneous T-cell lymphomas, neuroblastoma, prevention of squamous cell carcinoma in high-risk patients, rosacea, folliculitis, and pyoderma faciale [1,2].

The drug has low bioavailability, is highly lipophilic, and should be taken with a full glass of water and with a fat-containing meal. Depending on patient tolerance, the initial dose should be 0.5 mg/kg/day, and should then be gradually increased to 1.0 mg/kg/day for a 15- to 20-week course. The label-recommended cumulative dose, maintained in clinical trials, consensuses, and dermatological practice, ranges from 120–150 mg/kg [2,3].

Isotretinoin was synthesized in 1955; however, the way it works in acne vulgaris therapy is still not fully understood. ISO is metabolized in the liver by the cytochrome P-450 (CYP) microsomal enzyme system, principally by CYP2C8, CYP2C9, CYP3A4, and CYP2B6 isoenzymes, to three main metabolites: 4-oxo-isotretinoin, retinoic acid (tretinoin), and 4-oxo-retinoic acid (4-oxo-tretinoin). A hypothetical part of its action in acne treatment is binding to the retinoic acid nuclear receptors gamma (RAR-γ). Other known mechanisms of ISO action are inhibition of the production of cytokeratins 1, 10, and 14, filaggrin and matrix metalloproteinases (MMPs), and increases in cytokeratins 7, 13, and 19, laminin B1, and interleukin 1 (IL-1). The drug regulates gene expression, influencing nuclear transcription factors, which affect proliferation, differentiation, apoptosis, and cell renewal. There are also several underexplored actions, which can be presumably explained by its interaction with Forkhead Box Protein O1 (FOXO1). While the exact mechanism is unknown among patients suffering from acne, isotretinoin inhibits infundibular hyperkeratinization and the formation of comedones and induces apoptosis in sebocytes, which in turn decreases sebum production. Thus, 13-cis retinoic acid reduces the size of sebum ducts, making the microenvironment less hospitable to bacteria and alters immune mechanisms and chemotaxis of polymorphonuclears and monocytes [3,4,5].

Isotretinoin and its metabolites affect a substantial number of human cell types, causing different effects, both desirable and undesirable. Their impact on keratinocytes is responsible for mucocutaneous adverse effects, on hair follicle cells for the appearance of the telogen effluvium, on myocytes—creatine phosphokinase (CPK) release, and hepatocytes—an increase in homocysteine levels. It can affect neural crest cells, which may cause teratogenicity, or hippocampus cells, which can implicate reduction of hippocampal neurogenesis and depression. Thus, all women must use contraception agents. Other conditions that ISO can cause are inflammatory bowel disease by its interaction with intestinal epithelium, and dry eyes by the impact on meibomian cells [1,3].

Since isotretinoin’s introduction, 39 years have passed. The drug continues to be highly effective. In addition, its use has never been discontinued and it is highly effective, despite causing many side effects. Only a few countries have not yet approved it.

ISO is in the top 10 of the US Food and Drug Administration’s (FDA) database of drugs associated with reports of depression and suicide attempts. However, according to clinical studies, psychiatric side effects of this type appear in acne patients, reportedly regardless of whether they are taking isotretinoin or an antibiotic. ISO also causes liver test abnormalities in up to 15% of patients. However, the most frequently reported adverse reactions are those involving the skin and mucous membranes, central nervous system, musculoskeletal system, pregnancy, and eyes. Additionally, the most common side effect according to previous analysis is severe headache [1]. The range of side effects may vary depending on which isotretinoin preparation the patient will be taking. However, there is a lack of clinical trials evaluating different drugs containing isotretinoin as an active ingredient. Our work is a prelude to a potential future analysis of different drugs containing isotretinoin in relation to the range of possible side effects included in this meta-analysis.

During the COVID-19 pandemic, the perspective on acne completely changed. Widespread personal protective equipment (PPE) use, such as wearing masks, contributed to the coining of the new term—“maskne” or “mask-related acne”. It describes a specific subgroup of acne, associated with wearing reusable fabric on the face, which arises in a completely different pathomechanism. The appearance of skin lesions is influenced by an occlusive microenvironment that leads to dysbiosis of the microbiome due to lack of skin breathability, stickiness sensations, moisture saturation, lack of hygiene maintenance, increased temperature, perspiration and absorption of biological fluid of the nasal and oral orifices by the cotton mask. In addition, pressure may contribute to skin lesions [6]. According to a retrospective study, almost 80% of the participants reported acne severity, relapses, or first-time occurrence. Thus, PPE use triggered acne severity, which makes maskne a significant issue during the COVID-19 pandemic [7].

The purpose of this study is to establish the oral isotretinoin adverse events and their prevalence among human clinical trials.

## 2. Materials and Methods

### 2.1. Study Eligibility Criteria

The literature review and assessment were performed systematically by two authors (J.K. AND J.L.) in December 2021–February 2022. Three databases (PubMed, ClinicalTrials, and Cochrane Library) were searched using the following terms: “isotretinoin acne vulgaris” for published studies in English. Studies were included if they were human clinical trials published as full-text articles between 1980–2021 with no limitations regarding demographic characteristics, number, sex, age, race, or country origin of participants. Only trials gathering patients suffering from acne vulgaris and its variations were included. Drug intervention of incorporated studies concerned both monotherapy and combination therapies. Chosen research must have reported dosage and dosage schemes to be included. Studies were later excluded if they did not mention any information about adverse events (AEs). Eventual disagreements were solved through consensus.

### 2.2. Data Extraction

The data collection was performed independently by two reviewers (J.K. AND J.L.) with emphasis on the name or author of the study, year of publication, study population, study design, drug intervention, dosage, therapy duration, cumulative dose, and adverse events (AEs). Thus, biochemical deviations were also involved. At this stage, the authors decided to divide trials into intervention arms (IAs) to exclude subpopulations failing to meet the eligibility criteria and to separate different treatment schemes. Possible incompatibilities were elucidated via consensus.

### 2.3. Data Synthesis and Analysis

Synthesis of findings were in the form of a meta-analysis by two reviewers (J.K, J.L.) with emphasis on AEs grouping, cumulative dose calculation, and particular AEs prevalence calculation. Synonyms of AEs were merged into one during the first step. Second, the authors combined some similar (in terms of, e.g., pathogenesis or affected organ) AEs into groups, if it was possible. ISO cumulative dose calculation was a product of the daily dose and the duration of the course. If previously mentioned indicators were expressed in ranges, the authors used the mean of min and max values. Although, this solution can provide some limitations. AE prevalence was calculated by dividing the total number of patients across trials with a given AE by the total number at risk. It is vital to know that trials observed the different sets of AEs. Thus, if the trial did not mention a particular AE, the authors did not count the IA population. Eventual disagreements during synthesis were solved through consensus.

## 3. Results

### 3.1. Study Selection and Characteristics

To evaluate the certainty of evidence, the Grading of Recommendations—Assessment, Development and Evaluation (GRADE) was used across all the dominants: methodological limitations of the studies, indirectness, imprecision, inconsistency, and publication bias. The GRADE assessment was conducted for each AE group. Indirectness was mainly observed when significant differences in pharmacotherapy within one IA group occurred. Imprecision lowered the certainty of evidence when insufficient IAs were noticed (nAEs ≤ 3). A dose–response gradient was observed via Pearson’s correlation coefficient (PCC) [8,9,10].

**Figure 1:**
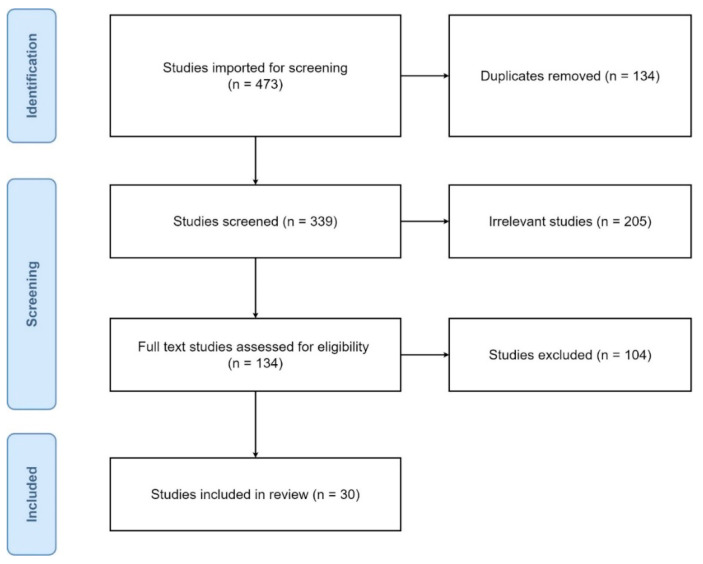
PRISMA flow diagram of literature search and selection process.

The primary outcome of interest was to collect results from articles concerning oral isotretinoin therapy and to summarize its clinical efficacy and adverse event profile with a placebo. However, because of the lack of placebo groups or insufficient data in this area, the basic form of data processing, meta-analysis, was replaced by single-arm meta-analysis. During the review, the authors identified 473 studies, of which 134 were assessed for full-text eligibility (Figure 1). Finally, 25 randomized controlled clinical trials (RCTs) and five open-label clinical trials provided 47 IAs. Results of the review are presented in Table 2 and Figure S1, providing a summary of the included studies and their course description. Among 30 studies, 3274 patients suffering from acne vulgaris were treated with ISO. The pharmacotherapy had different daily doses (ranging from 0.1–1.77 mg/kg/day) divided into two equal parts. However, in a substantial number of IAs, the daily dose was modified depending on study design and patient tolerance. Drugs were given mainly each day; however, some exceptions occurred: alternate-day administration (2 IAs), one week every 4 weeks administration (3 IAs), and 10 first days of the month administration (2 IAs). Therapy duration lasted from 8 weeks to ~8.3 months. Thus, the cumulative doses were also unequal, ranging from 11.2–309.8 mg/kg. The monotherapy of ISO was used in the majority of the studies except for four IAs using oral azithromycin and topical 1% clindamycin phosphate, two IAs using vitamin E, two IAs using vehicle gel, one IA using oral omega-3, one IA using pulsed dye laser (PDL) sessions, one IA using non-ablative fractional laser (NAFL) sessions, and one IA using topical agents (adapalene 0.1% gel and fusidic acid 2% cream).

After merging synonyms and assessing AEs into specific groups, 86 classifications were established: whole skin changes (dry skin, skin fragility, erythemal changes, sunburn, decreased sweating, dryness of other mucosal tissues, hair changes, hand changes, skin atrophy, flare of acne), ophthalmic changes (dry or irritated eyes, eye pain, vision changes, conjunctival changes), nasopharyngeal changes (dry nose, nasopharyngitis, UPTRI, epistaxis, cough), oral changes (dry lips, cheilitis, dry and sore mouth), mood and neurological changes (hearing changes, headache, mood changes, excessive thirst, decreased appetite, increased appetite, fatigue, and tiredness), MS changes (back pain, arthralgia, MS discomfort), GI changes (GI disorders, abdominal pain), infections (infections, herpes simplex, fever), other (Stevens–Johnson syndrome, morbilliform eruption, pyogenic granuloma, irregular cycle), liver function tests abnormalities (AST activity elevation, ALT activity elevation, GGT activity elevation, ALP activity elevation, serum total protein level elevation, serum albumin level elevation, LDH activity elevation, total bilirubin level elevation, direct bilirubin level elevation), lipid test abnormalities (total cholesterol level elevation, HDL level reduction, LDL level elevation, VLDL level elevation, triglycerides level elevation), blood count abnormalities (ERS elevation, total blood count abnormalities, RBC count abnormalities, reticulocytes count abnormalities, Hb level abnormalities, HCT abnormalities, WBC count abnormalities, neutrophiles count abnormalities, eosinophiles count abnormalities, basophils count abnormalities, monocytes count abnormalities, lymphocytes count abnormalities, platelets count abnormalities), urine test abnormalities (urine test abnormalities, urine specific gravity elevation, WBC count in urine elevation), kidney function test abnormalities (blood urea nitrogen level elevation, serum uric acid level elevation, serum CK activity elevation), serum glucose level abnormalities, calcium–phosphate regulation abnormalities (serum Ca level abnormalities, serum P level abnormalities, serum 25(OH)D level abnormalities), and semen test abnormalities.

### 3.2. Limitations

In our analysis, trial design was a major limitation. The lack of a placebo significantly affected results and reduced their certainty. In some cases, the limitation was the small population or number of IAs. Thus, obtained outcomes might not correspond to the results if the cohort was greater. Only trials published in English were included, excluding potentially relevant trials published in other languages. In addition, insufficient data about ISO cumulative doses completed by a mean value could be a limitation as well as the lack of brand name of administered drugs.

### 3.3. Certainty of Evidence

Based on the GRADE evaluation, certainty of registered AEs prevalence ranged from very low to high (5 high, 57 moderate, 16 low, and 10 very low). The main reasons for the low certainty were indirectness and imprecision. The most common indirectness was observed via high heterogeneity among ISO dosing, administration scheme, therapy duration, and biochemical markers reference ranges (especially liver enzymes activity, cholesterol level, and triglycerides level). In some cases, imprecision occurred when the numerous adverse events groups were insufficient (nAEs ≤ 3). Inconsistency among AE groups was not observed because data synthesis was based on numerical values, not means. Publication bias was not spotted by reviewers. Rarely have we observed dose–response gradients that increase the certainty. The positive correlation concerned dry skin (r, 0.41), dryness of other mucosal tissues (r, 0.99), dry nose (r, 0.54), epistaxis (r, 0.60), dry lips (r, 0.41), and arthralgia (r, 0.54).

## 4. Discussion

### 4.1. Whole Skin Changes

The common and less severe adverse events related to oral isotretinoin include mucocutaneous conditions such as xerosis, skin fragility, erythemal changes, and pruritus or rashes (Table 3). According to clinical, biochemical, and microscopic observations, systemic ISO therapy leads to a decline in skin sebum production and an increase in skin hydration, although it does not influence skin elasticity. This reaction combined with the reduction of the thickness of the stratum corneum and alteration of the cutaneous barrier is the most probable cause of the mucocutaneous changes [41,42].

13-cis retinoic acid causes telogen effluvium, which occasionally leads to coincidental androgenetic alopecia, and hair loss may continue. However, isotretinoin-dependent alopecia is the least common compared to other retinoids (8–10%) and is rather not dose-related. The studies included in the meta-analysis did not analyze the reversal of lesions after treatment, but if the alopecia was telogen effluvium, it most likely disappeared after treatment. Moreover, İslamoğlu et al. published a randomized controlled trial, where they support the hypothesis that Isotretinoin does not alter hair growth parameters in the short term of intake and low doses [43].

In addition, treatment with isotretinoin may result in the appearance of paronychia and excess granulation tissue in the nail furrows, manifesting as two patterns in the peri-nail location and in post-acne lesions. The topic is challenging because a review of the literature revealed few reports of these side effects [44].

### 4.2. Ophthalmic Changes

The ophthalmic changes caused by isotretinoin are strongly related to each other (Table 4). Dry or irritated eyes and inflammation of the eyelids may cause vision changes such as blurred vision, reduction of night vision, conjunctival changes, or subjective perception of eye pain. Scientists suspect that the possible cause of all the ophthalmic conditions may be atrophy of lacrimal and meibomian glands. Thus, the decrease in tear film quality leads to conjunctivitis, cornea opacity, and a reduction in corneal sensitivity [45].

Karalezli et al. in a prospective study from 2009 assessed that during 9 months of ISO treatment with a daily dose of 0.8 mg/kg, mean anaesthetized Schirmer test scores and tear breakup time decreased significantly during treatment (*p* < 0.001), which only confirms the thesis statement of isotretinoin effect on corneal sensitivity. In addition, a significant elevation of mean impression cytology scores, Ocular Surface Disease Index scores, and rose bengal staining scores during pharmacotherapy was observed (*p* < 0.05, *p* < 0.001, *p* < 0.001, respectively); blepharitis was seen in 36% of patients. However, all abnormal findings disappeared 1 month after the cessation of treatment. Thus, ocular changes are completely reversible and can be ameliorated by preventing dry eye. In addition, the varying incidence of symptoms may be due to a lack of patient education on how they can prevent these side effects by using mild cleansers, moisturizers, or lubricants from the first day of treatment [46].

Another trial searching for the long-term effects of isotretinoin (0.5–1.0 mg/kg/day) on macula ganglion cell complex thickness was performed by Demirok et al. and was published in 2017. It showed no significant effects of ISO on the thickness of the macula ganglion cell [47].

Ucak et al. published a trial in 2014, where they assessed possible toxic effects of the treatment with isotretinoin 0.5–2 mg/kg/day for 4–9 months on the retinal nerve fiber layer (RNFL) and ganglion cell layer (GCL), and the results showed a significant decrease in posttreatment lower temporal (TL) values [48].

Gediz et al. last year published results of a mean 7-month course of isotretinoin treatment. In the intervention group, subfoveal choroidal thickness and submacular choroidal vascularity index values were significantly higher than in the placebo group. However, they analyzed a small cohort of 25 patients and did not determine whether the choroidal lesions were reversible or not [49].

### 4.3. Nasopharyngeal Changes

Retinoids cause symptoms of dry nose and disturbed mucociliary clearance without affecting pulmonary function tests (Table 5). However, they are generally not severe, especially when isotretinoin is taken in low doses. In our study, we observed dose–response gradients concerning the appearance of dry nose, epistaxis, and dryness of other mucosal tissues. Mucosal side effects such as nasopharyngitis, UPTRI, epistaxis, and cough are directly attributable to mucosal dryness. Moisturizing the mucous membranes could decrease either the severity or prevalence of them [22].

### 4.4. Oral Changes

The most common side effects connected with isotretinoin therapy are mucocutaneous changes, especially dry lips (Table 6). Drug-induced cheilitis and excessive thirst are mainly caused by dryness of the mouth and lips. Oral changes are dose related and are completely reversible after completing therapy. In comparison to ophthalmic changes, they can be controlled by using mild cleansers, moisturizers, or lubricants from the first day of treatment [50].

### 4.5. Mood and Neurological Changes

Last year, Kemeriz et al. stated a hypothesis that reversible hearing loss during an oral isotretinoin course might be caused by hyperlipidemia, which leads to a decrease in cochlear oxygenation, direct toxicity on the cochlea, or a decrease in vascularization. Thus, research in this area should be extended [51].

Headaches are usually an intermittent and minor side effect and resolve quickly when treatment is discontinued (Table 7). However, it must be diagnosed carefully, while it can be symptomatic for other more serious conditions, e.g., pseudotumor cerebri [52].

Additionally, oral ISO seems to reduce hippocampal neurogenesis and alter the expression of serotonergic system components, while it is liposoluble and crosses the hemato-encephalic barrier. However, it was never further confirmed [42].

Ormerod et al. published in 2012 a prospective observational study with emphasis on isotretinoin’s influence on hippocampal-based learning in human subjects. Its performance stayed unchanged for delayed matching to sample (DMS), spatial recognition memory (SRM), and pattern recognition memory (PRM) tasks after 3–6 months of isotretinoin therapy. Instead, participants improved their speed on the PRM task and performance on the paired-associate learning (PAL) task, and those improvements were ISO dose related [53].

There is a hypothesis that neuropsychiatric manifestations, tiredness, and fatigue may occur secondary to hyperhomocysteinemia, vitamin B12, and folic acid deficiencies. Various studies have confirmed that acne therapy with isotretinoin causes a decrease in the serum level of folic acid and vitamin B12 and an increase in homocysteine levels. However, Ergun et al. in 2012 using a wide amount of tests stated that isotretinoin has no negative effect on attention, executive function, and mood [42,54,55,56,57,58].

Nevoralová et al. in their prospective study found no depressive symptoms or suicide risk caused by 9 months of isotretinoin 0.5 mg/kg/day treatment, whereas a reduction of depression signs was found in the Beck’s Depression Inventory, Version II (BDI-II). The hypothesis of isotretinoin improving depressive and anxiety symptoms and quality of life was confirmed in Kaymak et al., Bozdağ et al., Rehn et al., Ferahbas et al., and Rubinow et al. Clinically significant depression was no more prevalent in the isotretinoin group than in the conservative therapy group. The correlation between depression and isotretinoin can be only a rare unpredictable idiosyncratic side effect among patients with increased susceptibility to neuropsychiatric manifestations [59,60,61,62,63,64,65,66].

Karadag et al. reported that basal leptin levels were significantly lower in the group with acne compared to the control group, which may be a cause of increased appetite. However, some patients experienced decreased appetite as an adverse event after isotretinoin treatment. That is confirmed by the study of Cemil et al. who stated in contrast to Karadag et al. that leptin was increased after isotretinoin treatment [67,68].

### 4.6. Musculoskeletal Changes

The most common rheumatologic side effects of the ISO are musculoskeletal pain and arthralgia (Table 8). Other less common AEs are hyperostosis, extraspinal calcifications, enthesitis, arthritis, costochondritis, osteoporosis, growth retardation, premature epiphyseal closure in children, and gout can also occur, although the exact pathogenesis is unclear. Arthralgia may relate to isotretinoin detergent-like effects, alterations in the lysosomal membrane structure of cells, and the degeneration process in the synovial cells. ISO can also stimulate MMP-2 activity, causing membrane damage in the joints, and is suspected to render cells susceptible to mild traumas that normally would not cause injury [69].

A double-blind, randomized, multicenter study from 2015 among 358 children receiving isotretinoin demonstrates no effect on pediatric spine bone density after 6 months of treatment. In opposite, a prospective study by Leachman et al. from 1999 presented that bone density in young men (aged 17–25 years) after receiving oral isotretinoin for cystic acne was lower at all sites (spine, femoral neck, and Ward triangle) was considerably more variable at the spine even before treatment. A potential explanation could be a significant decrease within 5 days of treatment (*p* < 0.05) of markers of bone turnover, e.g., serum osteocalcin, the carboxyterminal propeptide of type I collagen, bone-specific alkaline phosphatase, the carboxyterminal telopeptide of type I collagen, and urine levels of calcium and hydroxyproline, serum calcium and parathyroid hormone, which were presented in a clinical trial of Kindmark et al. On the positive side is the fact that the abnormal levels of these markers returned to baseline values within 14 days [70,71,72].

### 4.7. Gastrointestinal Changes

A concerning side effect of isotretinoin is hypertriglyceridemia, which may cause pancreatitis. A well-known side effect of the drug is a toxic effect on the liver, whose failure can cause dyspeptic symptoms (Table 9) [73].

There have been concerns that oral use of 13-cis-retinoic acid may induce inflammatory bowel disease (IBD). However, two systematic reviews and meta-analyses concluded that there was no evidence for such an association [74,75].

The certainty of evidence indicating the presence of gastrointestinal lesions is moderate. Events relating to the gastrointestinal system are also not common and may be due to individual predisposition or improper oral drug intake.

### 4.8. Infections

Infections and associated fever are rare adverse reactions related to the use of isotretinoin (Table 10). A limitation to identifying these symptoms as related to the drug is that they could occur independently of acne treatment. In addition, there is a lack of studies that have proven an association by analyzing the pathomechanism by which isotretinoin could potentially increase the risk of infection.

### 4.9. Others

Stevens–Johnson Syndrome (SJS) is a life-threatening inflammatory mucocutaneous drug reaction, which usually begins with vague upper respiratory tract symptoms, such as fever, sore throat, chills, headaches, and malaise (Table 11). This is followed by the rapid onset of painful erosions of the mucous membranes, diffuse erythematous macules with purpuric, necrotic centers, and overlying blistering that often demonstrates a positive Nikolsky sign. Various theories have implicated both immunological and non-immunological pathophysiologic mechanisms of SJS, with the prevailing evidence suggesting that primary involvement of the immunologic response is mediated by memory cytotoxic T cells. Certain genetic predispositions may also increase the risk of patients developing SJS. Given this, it appeared in only one study, and in only 3.45% of patients. It is not a syndrome closely related to isotretinoin, but as it is a life-threatening reaction, it should always be considered in patients whose clinical condition is rapidly deteriorating. Similarly, the appearance of morbilliform eruption in three papers with a prevalence of 5.26% and pyogenic granuloma in two study arms with a prevalence of 0.19%, are related to the individual’s predisposition to develop the syndromes rather than a direct link between the drug and the drug reactions [76].

The hypothesis that acne treatment may cause possible depletion of ovarian reserve had been initiated by Remzi Abali et al. in 2013, who showed that isotretinoin caused a reduction of anti-Müllerian hormone (AMH) and increased atresia of follicles in rats. Further studies among omen suggested that isotretinoin could alter ovarian function by reduction of AMH and could cause a significant reduction of follicle stimulating hormone (FSH), luteinizing hormone (LH), and estradiol (E2). However, the controversy was elucidated in studies that detected no change in clinical trials of antral follicle count and ovarian volume, and it was proven that the change in hormone levels was reversible after the end of the treatment [77,78,79,80].

Additionally, in 2016, Levent Çinar et al. published a study in which they assessed the effect of systemic isotretinoin in a daily dose of 0.5–1.0 mg/kg up to 6 months on male fertility. No significant change in hormone levels was observed either; furthermore, all of the spermiogram parameters changed positively [81].

### 4.10. Laboratory Abnormalities

ISO therapy affects liver function tests and lipid profile, with a greater predilection to increase lipids than liver enzymes (Table 12). The findings of previous studies suggest that there is a small decrease in hepatic microsomal enzyme activity after ISO therapy and that the unwanted effects on lipid metabolism and liver function are unlikely to be due to hepatic microsomal enzyme induction [1]. In almost all cases, abnormalities in liver parameters and lipid profile resolved immediately after the end of therapy.

In half of the cases, the erythrocyte sedimentation rate was also increased. In addition, in some studies, increased proteinuria and specific gravity have been observed during ISO treatment. The cases in which abnormalities of kidney parameters were observed were causal and most likely depended on independent external factors.

## 5. Conclusions

This single-arm meta-analysis was conducted to highlight the effects of isotretinoin pharmacotherapy on human body function and to summarize the wide range of adverse effect profiles based on analysis of clinical trials since they first appeared in the databases. After synthesis, reported adverse events in included studies concerned: whole skin changes, ophthalmic changes, nasopharyngeal changes, oral changes, mood and neurological changes, musculoskeletal changes, gastrointestinal changes, infections, laboratory abnormalities, and other less common ones such as Stevens-Johnson syndrome, morbilliform eruption, pyogenic granuloma, and irregular cycle. Isotretinoin adverse events were mainly mucocutaneous conditions or conditions related to them such as dry skin, skin fragility, dermatitis, erythemal changes, xerosis, rashes, dry lips, dry and sore mouth, excessive thirst, cheilitis, and dry nose. Fortunately, they are all reversible and not severe. Teratogenicity can be avoided by contraception agents for the time of isotretinoin use, and those most severe are rare and represent individual responses for a drug. An understanding of the adverse events profile and its explanation to the patient increases the likelihood that the patient will comply with the recommended dosing regimen and will use additional treatment, which can alleviate or eliminate undesirable effects.

## Figures and Tables

**Table 1:** Isotretinoin—summary and structural formula [1].

Name	Other Names	Molecular Formula	Structure
Isotretinoin (ISO)	13-cis-Retinoic Acid, Accutane, Ro 4-3780, Roaccutane	C_20_H_28_O_2_	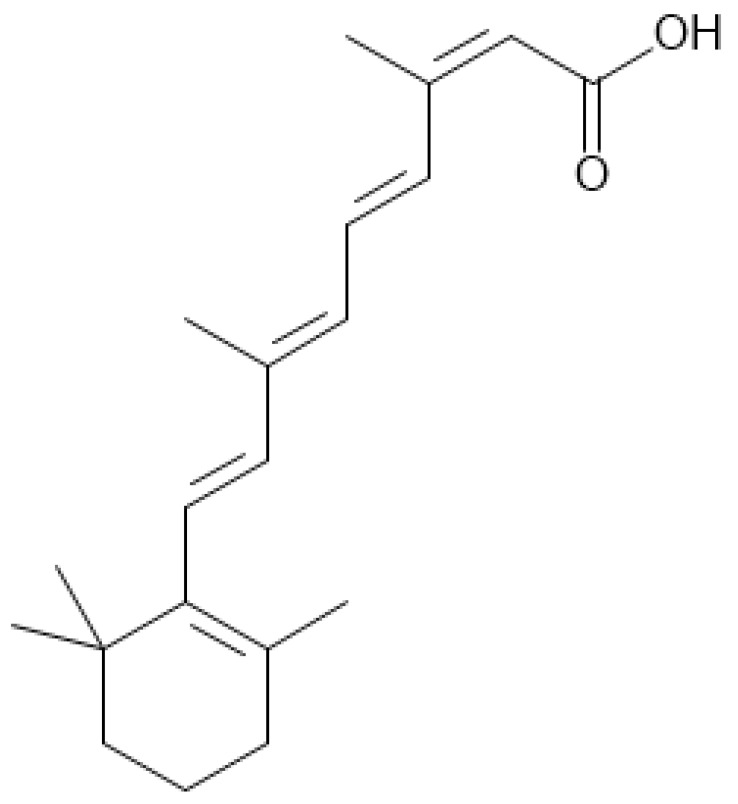

Structural formula made in “MedChem Designer™ version 5.5.0.11 64-bit edition” (Simulations Plus Inc., Lancaster, CA, USA).

**Table 2:** Summary of included studies: clinical adverse events in isotretinoin therapy.

Name/Author and Year of Publication	Population	Study Design	Drug Intervention and Scheme
Del Rosso et al., 2019 [11]	166	open-label CT	Lidose-ISO 0.5 mg/kg/day (4 weeks); 1.0 mg/kg/day (16 weeks); no treatment (104 weeks)
NCT00975143 [12]	925	RCT	ISO or CIP-ISO 0.5 mg/kg/day (2 weeks); 1.0 mg/kg/day (16 weeks)
Tan et al., 2014 [13]	133	RCT	ISO 0.5 mg/kg/day (4 weeks); 1.0 mg/kg/day (4 months) + vehicle gel
Li et al., 2020 [14]	23	RCT	ISO 0.5–0.75 mg/kg/day (8 weeks) + topical agents (adapalene 0.1% gel, fusidic acid 2% cream)
Xia et al., 2018 [15]	18	RCT	ISO 0.15–0.4 mg/kg/day (3 months) + 3 sessions of non-ablative fractional laser (NAFL)
Ibrahim et al., 2020 [16]	46	RCT	Group A: isotretinoin 0.25 mg/kg/day + 5 sessions of pulsed dry laser (PDL) (over 6 months); Group B: isotretinoin 0.5 mg/kg/day (over 6 months)
Rademaker et al., 2014 [17]	60	RCT	ISO 0.5 mg/kg/day (32 weeks)
Ahmad et al., 2015 [18]	58	RCT	ISO 0.5–1.0 mg/kg/day (3 months)
NCT01474590Tan [19]	133	RCT	ISO 0.5 mg/kg/day (4 weeks); ISO 1 mg/kg/day (16 weeks) + vehicle gel
Oprica et al., 2007 [20]	52	RCT	ISO 1.0 mg/kg/day (24 weeks)
Agarwal et al., 2011 [21]	120	RCT	Group A: ISO 1.0 mg/kg/day; Group B: ISO 1.0 mg/kg/alternate day; Group C: ISO 1.0 mg/kg/week/4 weeks (16 weeks); Group D: ISO 20 mg/every alternate day (16 weeks); for all groups oral azithromycin 500 mg/day/3 days a week (3 weeks) + topical 1% clindamycin phosphate/2 × day
Gorpelioglu et al., 2010 [22]	40	open-label CT	ISO 0.5–1.0 mg/kg/day (at least 3 months)
Akman et al., 2007 [23]	66	RCT	Group A: ISO 0.5 mg/kg/day/first 10 days of each month (6 months); Group B: ISO 0.5 mg/kg/day (1 month); ISO 0.5 mg/kg/day/the first 10 days of each month (5 months); Group C: ISO 0.5 mg/kg/day (6 months)
Lee et al., 2011 [24]	60	RCT	Group A: ISO 0.5–0.7 mg/kg/day; Group B: ISO 0.25–0.4 mg/kg/day; Group C: ISO 0.5–0.7 mg/kg/day for 1 week of every 4 weeks (24 weeks)
Kus et.al., 2005 [25]	60	RCT	Group A: ISO 1.0 mg/kg/day; Group B: ISO 1.0 mg/kg/day + vit. E 800 IU/day (16 weeks)
Kaymak et al., 2006 [26]	41	open-label CT	ISO 0.5–0.75 mg/kg/day, for 1 week every 4 weeks (6 months)
DiGiovanna et al., 2004 [27]	217	RCT	ISO 1.0 mg/kg/day (16–20 weeks)
Strauss et al., 2000 [28]	140	RCT	Group A: ISO 1.0 mg/kg/day; Group B: ISO 1.0 mg/kg/day + 800 IU/day vit. E (20 weeks)
Hermes et al., 1998 [29]	94	open-label CT	ISO ~0.43 mg/kg/day (~8.3 months)
Lin et al., 1999 [30]	18	RCT	ISO 10 mg/day (3 months)
Strauss et al., 2001 [31]	300	RCT	ISO 1.0 mg/kg/day (20 weeks)
Strauss et al., 1984 [32]	150	RCT	Group A: ISO 0.1 mg/kg/day; Group B: ISO 0.5 mg/kg/day; Group C: ISO 1.0 mg/kg/day (20 weeks)
Lester et al., 1985 [33]	15	RCT	ISO 0.5–2.0 mg/kg/day (16 weeks)
Goldstein et al., 1982 [34]	28	RCT	ISO 1.0 mg/kg/day (16 weeks)
Jones et al., 1983 [35]	76	RCT	ISO 0.1 mg/kg/day; Group B: ISO 0.5 mg/kg/day; Group C: ISO 1.0 mg/kg/day (16 weeks)
Blasiak et al., 2013 [36]	116	RCT	Group A: ISO mean cumulative dose < 220 mg/kg (5.8 months); Group B: ISO mean cumulative dose > 220 mg/kg (6.5 months)
Mirnezami et al., 2018 [37]	118	RCT	Group A: ISO 0.5 mg/kg/day; Group B: ISO 0.5 mg/kg/day + oral omega-3 1.0 g/day (16 weeks)
Kanigsberg et al., 1983 [38]	33	open-label CT	ISO 0.5–1.0 mg/kg/day (16 weeks)
Farrell et al., 1980 [39]	14	RCT	ISO 0.1–1.0 mg/kg/day (12 weeks)
Meeren et al., 1983 [40]	58	RCT	Group A: ISO 0.5 mg/kg/day; Group B: ISO 1.0 mg/kg/day (24 weeks)

Abbreviations: CT, clinical trial; RCT, randomized controlled trial; ISO, isotretinoin. Among selected articles, 124 particular AEs were extracted and included: “dry skin”, “skin irritation”, “skin fragility”, “dermatitis”, “facial dermatitis”, “eczema”, “erythematous eruption”, “erythema”, “bruising of the skin”, “xerosis”, “desquamation”, “localized exfoliation”, “rashes”, “pruritus”, “sunburn”, “fissuring”, “flushing”, “crusting of lesions”, “decreased sweating”, “facial dryness”, “facial redness”, “dry eyes”, “irritated eyes”, “visual acuity reduced”, “reduced night vision”, “blurred vision”, “increased photosensitivity”, “conjunctivitis”, “eye pain”, “xerophthalmia”, “pterygium”, “photophobia”, “dry nose”, “nasal crusting”, “nasopharyngitis”, “epistaxis”, “nasal bleeding”, “upper respiratory tract infection (UPTRI)”, “dry lips”, “dry mouth”, “chapped lips”, “peeling lips”, “sore mouth”, “cheilitis”, “angular stomatitis”, “hearing changes”, “hand dryness”, “peeling of fingertip skin”, “tender fingerprints”, “nail changes”, “body dryness”, “dryness of other mucosal tissues”, “headache”, “back pain”, “abdominal pain”, “arthralgia”, “musculoskeletal (MS) discomfort”, “gastrointestinal (GI) upset”, “nausea”, “vomiting”, “fatigue and tiredness”, “infections”, “cough”, “irregular cycle”, “pyogenic granuloma”, “dry hair”, “white hair”, “hair loss”, “alopecia”, “Stevens-Johnson syndrome”, “depressed mood, insomnia and hallucination”, “suicidal ideation”, “nervousness”, “excessive thirst”, “decreased appetite”, “increased appetite”, “fever”, “morbilliform eruption”, “herpes simplex”, “skin atrophy”, “flare of acne”, “liver enzymes activity elevation”, “AST (aspartate aminotransferase) activity elevation”, “ALT (alanine transaminase) activity elevation”, “GGT (gamma-glutamyl transferase) activity elevation”, “ALP (alkaline phosphatase) activity elevation”, “serum total protein level elevation”, “serum albumin level elevation”, “LDH (lactic-dehydrogenase) activity elevation”, “total bilirubin level elevation”, “direct bilirubin level elevation”, “total cholesterol level elevation”, “HDL (high-density lipoprotein) level reduction”, “LDL (low-density lipoprotein) level elevation”, “VLDL (very low-density lipoprotein) level elevation”, “triglycerides level elevation”, “ERS (erythrocytes sedimentation rate) elevation”, “total blood count abnormalities”, “RBC (red blood cells) count abnormalities”, “reticulocytes count abnormalities”, “Hb (hemoglobin) level abnormalities”, “HCT (hematocrit) abnormalities”, “WBC (white blood cells) count abnormalities”, “neutrophiles count abnormalities”, “eosinophiles count abnormalities”, “basophils count abnormalities”, “monocytes count abnormalities”, “lymphocytes count abnormalities”, “platelets count abnormalities”, “urine test abnormalities”, “urine specific gravity elevation”, “WBC count in urine elevation”, “blood urea nitrogen level elevation”, “serum uric acid level elevation”, “serum CK (creatine kinase) activity elevation”, “serum glucose level abnormalities”, “serum Ca (calcium) level abnormalities”, “serum P (phosphate) level abnormalities”, “serum 25(OH)D (25-hydroxyvitamin D) level abnormalities”, and “semen test abnormalities”.

**Table 3:** Summary of ISO therapy adverse events: whole skin changes.

Outcomes	Number of Participants (IAs)	Prevalence (%)	Certainty of the Evidence (GRADE)
Dry skin	3228 (45 IAs)	1584 (49.07%)	⨁⨁⨁⨁ high due to dose-response gradient, however indirectness present
Skin fragility	235 (7 IAs)	65 (27.66%)	⨁⨁⨁◯ moderate due to indirectness
Erythemal changes	2350 (33 IAs)	653 (27.79%)	⨁⨁⨁◯ moderate due to indirectness
Sunburn	141 (3 IAs)	17 (12.06%)	⨁⨁◯◯ low due to indirectness and imprecision
Decreased sweating	15 (1 IA)	1 (6.67%)	⨁◯◯◯ very low due to imprecision
Dryness of other mucosal tissues	238 (6 IAs)	70 (29.41%)	⨁⨁⨁⨁ high due to dose-response gradient, however indirectness present
Hair changes	837 (20 IAs)	149 (17.90%)	⨁⨁⨁◯ moderate due to indirectness
Hand changes	692 (18 IAs)	171 (24.71%)	⨁⨁⨁◯ moderate due to indirectness
Skin atrophy	58 (2 IAs)	2 (3.45%)	⨁⨁◯◯ low due to indirectness and imprecision
Flare of acne	96 (4 IAs)	46 (47.92%)	⨁⨁⨁◯ moderate due to indirectness

Abbreviations: IA, intervention arm; GRADE, Grading of Recommendation—Assessment, Development and Evaluation. Symbols are used to describe certainty in evidence profiles. High certainty: ⨁⨁⨁⨁; moderate certainty: ⨁⨁⨁◯; low certainty: ⨁⨁◯◯; very low certainty: ⨁◯◯◯.

**Table 4:** Summary of ISO therapy adverse events: ophthalmic changes.

Outcomes	Number of Participants (IAs)	Prevalence (%)	Certainty of the Evidence (GRADE)
Dry or irritated eyes	2244 (27 IAs)	603 (26.87%)	⨁⨁⨁◯ moderate due to indirectness
Eye pain	169 (4 IAs)	29 (17.16%)	⨁⨁⨁◯ moderate due to indirectness
Vision changes	1313 (10 IAs)	72 (5.48%)	⨁⨁⨁◯ moderate due to indirectness
Conjunctival changes	545 (11 IAs)	125 (22.49%)	⨁⨁⨁◯ moderate due to indirectness

Abbreviations: IA, intervention arm; GRADE, Grading of Recommendation—Assessment, Development and Evaluation. Symbols are used to describe certainty in evidence profiles. Moderate certainty: ⨁⨁⨁◯.

**Table 5:** Summary of ISO therapy adverse events: nasopharyngeal changes.

Outcomes	Number of Participants (IAs)	Prevalence (%)	Certainty of the Evidence (GRADE)
Dry nose	860 (18 IAs)	346 (40.23%)	⨁⨁⨁⨁ high due to dose-response gradient, however indirectness present
Nasopharyngitis	1543 (8 IAs)	205 (13.29%)	⨁⨁⨁◯ moderate due to indirectness
UPTRI	1058 (2 IAs)	52 (4.91%)	⨁⨁◯◯ low due to indirectness and imprecision
Epistaxis	2250 (30 IAs)	536 (23.82%)	⨁⨁⨁⨁ high due to dose-response gradient, however indirectness present
Cough	58 (1 IAs)	2 (3.45%)	⨁◯◯◯ very low due to imprecision

Abbreviations: IA, intervention arm; GRADE, Grading of Recommendation—Assessment, Development and Evaluation; UPTRI, upper respiratory tract infection. Symbols are used to describe certainty in evidence profiles. High certainty: ⨁⨁⨁⨁; moderate certainty: ⨁⨁⨁◯; low certainty: ⨁⨁◯◯; very low certainty: ⨁◯◯◯.

**Table 6:** Summary of ISO therapy adverse events: oral changes.

Outcomes	Number of Participants (IAs)	Prevalence (%)	Certainty of the Evidence (GRADE)
Dry lips	2309 (30 IAs)	1344 (58.21%)	⨁⨁⨁⨁ high due to dose-response gradient, however indirectness present
Cheilitis	2528 (27 IAs)	1049 (41.50%)	⨁⨁⨁◯ moderate due to indirectness
Dry and sore mouth	726 (21 IAs)	275 (37.88%)	⨁⨁⨁◯ moderate due to indirectness
Excessive thirst	393 (10 IAs)	138 (35.11%)	⨁⨁⨁◯ moderate due to indirectness

Abbreviations: IA, intervention arm; GRADE, Grading of Recommendation—Assessment, Development and Evaluation. Symbols are used to describe certainty in evidence profiles. High certainty: ⨁⨁⨁⨁; moderate certainty: ⨁⨁⨁◯.

**Table 7:** Summary of ISO therapy adverse events: mood and neurological changes.

Outcomes	Number of Participants (IAs)	Prevalence (%)	Certainty of the Evidence (GRADE)
Hearing changes	116 (2 IAs)	2 (1.72%)	⨁⨁◯◯ low due to indirectness and imprecision
Headache	1967 (17 IAs)	207 (10.52%)	⨁⨁⨁◯ moderate due to indirectness
Mood changes	521 (11 IAs)	53 (10.17%)	⨁⨁⨁◯ moderate due to indirectness
Decreased appetite	141 (3 IAs)	23 (16.31%)	⨁⨁◯◯ low due to indirectness and imprecision
Increased appetite	156 (4 IAs)	6 (3.85%)	⨁⨁⨁◯ moderate due to indirectness
Fatigue and tiredness	543 (16 IAs)	132 (24.31%)	⨁⨁⨁◯ moderate due to indirectness

Abbreviations: IA, intervention arm; GRADE, Grading of Recommendation—Assessment, Development and Evaluation. Symbols are used to describe certainty in evidence profiles. Moderate certainty: ⨁⨁⨁◯; low certainty: ⨁⨁◯◯.

**Table 8:** Summary of ISO therapy adverse events: musculoskeletal changes.

Outcomes	Number of Participants (IAs)	Prevalence (%)	Certainty of the Evidence (GRADE)
Back pain	1500 (4 IAs)	294 (19.60%)	⨁⨁⨁◯ moderate due to indirectness
Arthralgia	1716 (15 IAs)	297 (17.31%)	⨁⨁⨁⨁ high due to dose-response gradient, however indirectness present
MS discomfort	1717 (20 IAs)	216 (12.58%)	⨁⨁⨁◯ moderate due to indirectness

Abbreviations: IA, intervention arm; GRADE, Grading of Recommendation—Assessment, Development and Evaluation; MS, musculoskeletal. Symbols are used to describe certainty in evidence profiles. High certainty: ⨁⨁⨁⨁; moderate certainty: ⨁⨁⨁◯.

**Table 9:** Summary of ISO therapy adverse events: gastrointestinal changes.

Outcomes	Number of Participants (IAs)	Prevalence (%)	Certainty of the Evidence (GRADE)
GI disorders	257 (5 IAs)	25 (9.73%)	⨁⨁⨁◯ moderate due to indirectness
Abdominal pain	555 (9 IAs)	51 (9.19%)	⨁⨁⨁◯ moderate due to indirectness

Abbreviations: IA, intervention arm; GRADE, Grading of Recommendation—Assessment, Development and Evaluation; GI, gastrointestinal. Symbols are used to describe certainty in evidence profiles. Moderate certainty: ⨁⨁⨁◯.

**Table 10:** Summary of ISO therapy adverse events: infections.

Outcomes	Number of Participants (IAs)	Prevalence (%)	Certainty of the Evidence (GRADE)
Infections	29 (1 IA)	11 (37.93%)	⨁◯◯◯ very low due to imprecision
Herpes simplex	15 (1 IA)	0 (0.00%)	⨁◯◯◯ very low due to imprecision
Fever	141 (3 IAs)	8 (5.67%)	⨁⨁◯◯ low due to indirectness and imprecision

Abbreviations: IA, intervention arm; GRADE, Grading of Recommendation—Assessment, Development and Evaluation. Symbols are used to describe certainty in evidence profiles. Low certainty: ⨁⨁◯◯; very low certainty: ⨁◯◯◯.

**Table 11:** Summary of ISO therapy adverse events: other.

Outcomes	Number of Participants (IAs)	Prevalence (%)	Certainty of the Evidence (GRADE)
Stevens-Johnson syndrome	58 (1 IA)	2 (3.45%)	⨁◯◯◯ very low due to imprecision
Morbilliform eruption	133 (3 IAs)	7 (5.26%)	⨁⨁◯◯ low due to indirectness and imprecision
Pyogenic granuloma	1058 (2 IAs)	2 (0.19%)	⨁⨁◯◯ low due to indirectness and imprecision
Irregular cycle	15 (1 IA)	0 (0.00%)	⨁◯◯◯ very low due to imprecision

Abbreviations: IA, intervention arm; GRADE, Grading of Recommendation—Assessment, Development and Evaluation. Symbols are used to describe certainty in evidence profiles. Low certainty: ⨁⨁◯◯; very low certainty: ⨁◯◯◯.

**Table 12:** Summary of ISO therapy adverse events: laboratory abnormalities.

Outcomes	Number of Participants (IAs)	Prevalence (%)	Certainty of the Evidence (GRADE)
Liver function tests abn	2018 (40 IAs)	99 (4.91%)	⨁⨁⨁◯ moderate due to indirectness
AST ↑	1768 (38 IAs)	77 (4.36%)	⨁⨁⨁◯ moderate due to indirectness
ALT ↑	1692 (35 IAs)	50 (2.96%)	⨁⨁⨁◯ moderate due to indirectness
GGT ↑	579 (6 IAs)	24 (4.15%)	⨁⨁⨁◯ moderate due to indirectness
ALP ↑	1034 (17 IAs)	8 (0.77%)	⨁⨁⨁◯ moderate due to indirectness
Total protein ↑	321 (7 IAs)	16 (4.98%)	⨁⨁⨁◯ moderate due to indirectness
Albumin ↑	188 (5 IAs)	0 (0.00%)	⨁⨁◯◯ low due to indirectness and imprecision
LDH ↑	621 (8 IAs)	58 (9.34%)	⨁⨁⨁◯ moderate due to indirectness
Total bilirubin ↑	278 (9 IAs)	2 (0.72%)	⨁⨁⨁◯ moderate due to indirectness
Direct bilirubin ↑	90 (4 IAs)	1 (1.11%)	⨁⨁◯◯ low due to indirectness and imprecision
Lipid test abn	2010 (39 IAs)	290 (14.43%)	⨁⨁⨁◯ moderate due to indirectness
Total cholesterol ↑	1927 (37 IAs)	142 (7.37%)	⨁⨁⨁◯ moderate due to indirectness
HDL ↓	508 (13 IAs)	83 (16.34%)	⨁⨁⨁◯ moderate due to indirectness
LDL ↑	367 (10 IAs)	87 (23.71%)	⨁⨁⨁◯ moderate due to indirectness
VLDL ↑	213 (4 IAs)	59 (27.70%)	⨁⨁◯◯ low due to indirectness and imprecision
Triglycerides ↑	2000 (39 IAs)	206 (10.30%)	⨁⨁⨁◯ moderate due to indirectness
Blood count abn	1062 (17 IAs)	21 (1.98%)	⨁⨁⨁◯ moderate due to indirectness
RBC abn	1054 (16 IAs)	0 (0.00%)	⨁⨁⨁◯ moderate due to indirectness
Reticulocytes abn	155 (4 IAs)	0 (0.00%)	⨁⨁◯◯ low due to indirectness and imprecision
Hb abn	1054 (16 IAs)	0 (0.00%)	⨁⨁⨁◯ moderate due to indirectness
HCT abn	1054 (16 IAs)	0 (0.00%)	⨁⨁⨁◯ moderate due to indirectness
WBC abn	1054 (16 IAs)	18 (1.71%)	⨁⨁⨁◯ moderate due to indirectness
Neutrophiles abn	593 (7 IAs)	3 (0.51%)	⨁⨁⨁◯ moderate due to indirectness
Eosinophiles abn	593 (7 IAs)	0 (0.00%)	⨁⨁⨁◯ moderate due to indirectness
Basophiles abn	593 (7 IAs)	3 (0.51%)	⨁⨁⨁◯ moderate due to indirectness
Monocytes abn	593 (7 IAs)	0 (0.00%)	⨁⨁⨁◯ moderate due to indirectness
Lymphocytes abn	593 (7 IAs)	0 (0.00%)	⨁⨁⨁◯ moderate due to indirectness
Platelets abn	1062 (17 IAs)	11 (1.04%)	⨁⨁⨁◯ moderate due to indirectness
ERS ↑	14 (1 IAs)	7 (50.00%)	⨁◯◯◯ very low due to imprecision
Urine test abn	296 (9 IAs)	16 (5.41%)	⨁⨁⨁◯ moderate due to indirectness
Specific gravity ↑	141 (3 IAs)	16 (11.35%)	⨁⨁◯◯ low due to indirectness and imprecision
WBC ↑	141 (3 IAs)	11 (7.80%)	⨁⨁◯◯ low due to indirectness and imprecision
Kidney function test abn	1286 (9 IAs)	55 (3.66%)	⨁⨁⨁◯ moderate due to indirectness
Blood urea nitrogen ↑	321 (7 IAs)	0 (0.00%)	⨁⨁⨁◯ moderate due to indirectness
Uric acid ↑	293 (6 IAs)	0 (0.00%)	⨁⨁⨁◯ moderate due to indirectness
CK ↑	1463 (9 IAs)	55 (3.76%)	⨁⨁⨁◯ moderate due to indirectness
Glucose abn	361 (8 IAs)	0 (0.00%)	⨁⨁⨁◯ moderate due to indirectness
Calcium-phosphate abn	231 (2 IAs)	0 (0.00%)	⨁◯◯◯ very low due to imprecision
Ca abn	231 (2 IAs)	0 (0.00%)	⨁⨁◯◯ low due to indirectness and imprecision
P abn	231 (2 IAs)	0 (0.00%)	⨁⨁◯◯ low due to indirectness and imprecision
25(OH)D abn	217 (1 IAs)	0 (0.00%)	⨁◯◯◯ very low due to imprecision
Semen test abn	28 (1 IAs)	0 (0.00%)	⨁◯◯◯ very low due to imprecision

Abbreviations: abn, abnormalities; ↑, level elevation or activity elevation; ↓, level reduction; AST, aspartate aminotransferase; ALT, alanine transaminase; GGT, gamma-glutamyl transferase; ALP, alkaline phosphatase; LDH, lactic-dehydrogenase; HDL, high-density lipoprotein; LDL, low-density lipoprotein; VLDL, very low-density lipoprotein; ERS, erythrocytes sedimentation rate; RBC, red blood cells; Hb, hemoglobin; HCT, hematocrit; WBC, white blood cells; CK, creatine kinase; Ca, calcium; P, phosphate; 25(OH)D, 25-hydroxyvitamin D; IA, intervention arm; GRADE, Grading of Recommendation֫—Assessment, Development and Evaluation. Symbols are used to describe certainty in evidence profiles. Moderate certainty: ⨁⨁⨁◯; low certainty: ⨁⨁◯◯; very low certainty: ⨁◯◯◯.

## Data Availability

Not applicable.

## Supplementary Materials

The following supporting information can be downloaded at: https://www.mdpi.com/article/10.3390/ijerph19116463/s1, Figure S1. Isotretinoin therapy adverse events prevalence: (A) Isotretinoin therapy adverse events; (B) Isotretinoin therapy adverse events: oral changes; (C) Isotretinoin therapy adverse events: whole skin changes; (D) Isotretinoin therapy adverse events: nasopharyngeal changes; (E) Isotretinoin therapy adverse events: ophthalmic changes; (F) Isotretinoin therapy adverse events: musculoskeletal changes; (G) Isotretinoin therapy adverse events: laboratory abnormalities; (H) Isotretinoin therapy adverse events: mood and neurological changes; (I) Isotretinoin therapy adverse events: gastrointestinal changes. Abbreviation: UPTRI, Upper Respiratory Tract Infection; MS, musculoskeletal; abn, abnormalities; GI, gastrointestinal.
