# Supplementary material for: Adverse Events in Isotretinoin Therapy: A Single-Arm Meta-Analysis
Source: Int J Environ Res Public Health. 2022 May 26;19(11):6463. doi: 10.3390/ijerph19116463 (PMC9180136; doi:10.3390/ijerph19116463)
Supplement: Supplementary file 1 [file ijerph-19-06463-s001.zip › ijerph-1690260-supplementary.pdf]

International Journal of
Environmental Research and Public Health

MDPI

Meta-analysis

Supplementary Materials: Adverse Events in Isotretinoin Therapy: A Single-Arm Meta-Analysis

Jan Kapała [1,*], Julia Lewandowska [1], Waldemar Placek [2] and Agnieszka Owczarczyk-Saczonek [2]

[1] Medical Faculty, University of Warmia and Mazury in Olsztyn, 10-719 Olsztyn, Poland; julia.lewandowska.1@student.uwm.edu.pl
[2] Department of Dermatology Sexually Transmitted Diseases and Clinical Immunology, University of Warmia and Mazury in Olsztyn, 10-719 Olsztyn, Poland; w.placek@wp.pl (W.P.); agnieszka.owczarczyk@uwm.edu.pl (A.O.-S.)
* Correspondence: jan.kapala@student.uwm.edu.pl

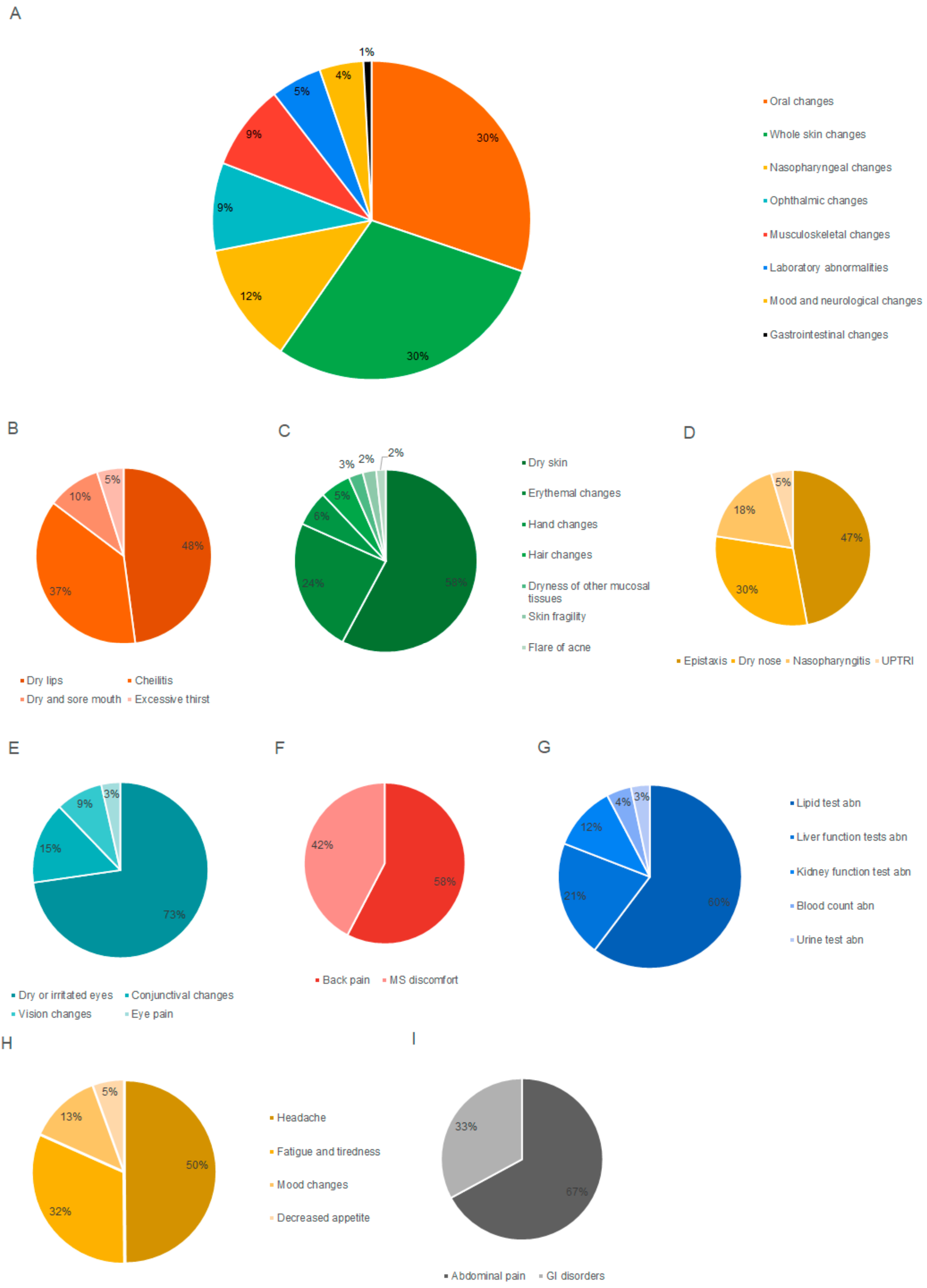
A
1%
4%
5%
9%
9%
12%
30%
30%
Oral changes
Whole skin changes
Nasopharyngeal changes
Ophthalmic changes
Musculoskeletal changes
Laboratory abnormalities
Mood and neurological changes
Gastrointestinal changes
B
5%
10%
48%
37%
Dry lips
Cheilitis
Dry and sore mouth
Excessive thirst
C
3%
2%
2%
5%
6%
24%
58%
Dry skin
Erythemal changes
Hand changes
Hair changes
Dryness of other mucosal tissues
Skin fragility
Flare of acne
D
5%
18%
47%
30%
Epistaxis
Dry nose
Nasopharyngitis
UPTRI
E
3%
9%
15%
73%
Dry or irritated eyes
Conjunctival changes
Vision changes
Eye pain
F
42%
58%
Back pain
MS discomfort
G
4%
3%
12%
21%
60%
Lipid test abn
Liver function tests abn
Kidney function test abn
Blood count abn
Urine test abn
H
5%
13%
50%
32%
Headache
Fatigue and tiredness
Mood changes
Decreased appetite
I
33%
67%
Abdominal pain
GI disorders

Figure S1. Isotretinoin therapy adverse events prevalence: (A) Isotretinoin therapy adverse events; (B) Isotretinoin therapy adverse events: oral changes; (C) Isotretinoin therapy adverse events: whole skin changes; (D) Isotretinoin therapy adverse events: nasopharyngeal changes; (E) Isotretinoin therapy adverse events: ophthalmic changes; (F) Isotretinoin therapy adverse events: musculoskeletal changes; (G) Isotretinoin therapy adverse events: laboratory abnormalities; (H) Isotretinoin therapy adverse events: mood and neurological changes; (I) Isotretinoin therapy adverse events: gastrointestinal changes. Abbreviation: UPTRI, Upper Respiratory Tract Infection; MS, musculoskeletal; abn, abnormalities; GI, gastrointestinal.
